# Normative Modeling Reveals Age‐Atypical Cortical Thickness Differences Between Hepatic Steatosis and Fibrosis in Non‐Alcoholic Fatty Liver Disease

**DOI:** 10.1002/brb3.70466

**Published:** 2025-04-07

**Authors:** Minchul Kim, Inpyeong Hwang, Kyu Sung Choi, Junhyeok Lee, Minjung Ryu, Jung Hyun Park, Joon Ho Moon

**Affiliations:** ^1^ Department of Radiology, Samsung Kangbuk Hospital Sungkyunkwan University School of Medicine Seoul South Korea; ^2^ Department of Radiology Seoul National University Hospital and Seoul National University College of Medicine Seoul South Korea; ^3^ Artificial Intelligence Collaborative Network Department of Radiology Seoul National University Hospital Seoul South Korea; ^4^ Department of Radiology, Interdisciplinary Program in Cancer Biology Seoul National University College of Medicine Seoul South Korea; ^5^ Department of Radiology Seoul Metropolitan Government Seoul National University Boramae Medical Center Seoul South Korea; ^6^ Department of Internal Medicine Seoul National University Bundang Hospital Seongnam South Korea

**Keywords:** brain age, cortical thickness, liver–brain axis, non‐alcoholic fatty liver disease, normative modeling

## Abstract

**Objectives:**

To investigate individual variations and outliers in cortical thickness among non‐alcoholic fatty liver disease (NAFLD) patients, ranging from hepatic steatosis to fibrosis, using neuroanatomical normative modeling.

**Materials and Methods:**

A cross‐sectional study with 2637 health check‐up subjects was conducted. Among NAFLD patients, hepatic steatosis (*n* = 556) and fibrosis (*n* = 57) were determined by hepatic steatosis index and fibrosis‐4 score, respectively. Cortical thickness in 148 different brain regions was assessed using T1‐weighted MRI scans. A publicly available neuroanatomical normative model analyzed cortical thickness distributions with data from around 58,000 participants. The hierarchical Bayesian regression was used to estimate cortical thickness deviation for each region, taking age, sex, and sites into account. On the basis of a normal adaptation set, *Z*‐scores below −1.96 or above +1.96 per region were classified as outliers. The total outlier count (tOC) was then calculated to quantify regional heterogeneity. Mass univariate analysis was conducted to compare steatosis and fibrosis groups, and the spatial patterns of regional heterogeneity were qualitatively analyzed.

**Results:**

Patients with hepatic fibrosis had a higher number of positive outlier regions (mean 6.3 ± 10.3) than hepatic steatosis (mean 4.2 ± 6.2, *p* = 0.02). Mass univariate group difference testing of 148 brain regions revealed patients with hepatic fibrosis had 6 cortical areas thicker than hepatic steatosis. Two groups showed shared regional heterogeneity in the temporal cortex.

**Conclusion:**

Distinct brain atrophy patterns were observed in NAFLD patients compared to the normal group, with more frequent temporal cortex outliers in both hepatic steatosis and fibrosis. Hepatic fibrosis showed slightly increased cortical thickness relative to steatosis.

## Graphical abstract

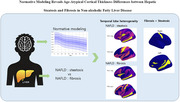

**Abbreviations**
AFLD: alcoholic fatty liver disease
Fib‐4: fibrosis‐4 score
HSI: hepatic steatosis index
NAFLD: non‐alcoholic fatty liver disease
tOC: total outlier count
tOC‐neg: total negative outlier count
tOC‐pos: total positive outlier count

## 1 Introduction

Hepatic steatosis refers to the accumulation of neutral lipid droplets in the hepatocyte cytoplasm (Peng et al. 2020). There are two main types of hepatic steatosis: alcoholic fatty liver disease (AFLD) and non‐alcoholic fatty liver disease (NAFLD), and they may coexist (Idalsoaga et al. 2020). Although both conditions share some similarities in pathology and progression (Åberg and Färkkilä 2020; Tarantino and Citro 2024), this study focuses on NAFLD to minimize confounding effects from alcohol‐related brain alterations (Park et al. 2021). NAFLD is frequently associated with obesity, insulin resistance, and dyslipidemia (Karanjia et al. 2016). Currently, around 25% of NAFLD patients are estimated to have non‐alcoholic steatohepatitis (NASH), which is characterized by excessive liver fat accumulation and hepatic inflammation (Peng et al. 2020), and patients with NASH at baseline showed a mean annual fibrosis progression rate of 0.09 (Younossi et al. 2016).

Extrahepatic manifestations of chronic liver diseases have recently gained more attention in both hepatology and neuroscience. The shared risk factors and mechanisms between NAFLD and brain damage raised a growing interest in the liver–brain axis (Jiang et al. 2023). Myriads of previous studies reported hepatic steatosis and fibrosis are associated with decreased brain volume in widespread cortical, subcortical, and cerebellar areas, implying heterogeneous brain involvement among subjects (Basu et al. 2022; Chen et al. 2021; Parikh et al. 2023; Weinstein et al. 2018, 2024). For instance, a study using data from the Framingham cohort found that NAFLD was associated with a smaller overall cerebral volume, implying an advancement of 4.2 years in brain aging (Weinstein et al. 2018). However, although previous studies have discussed general brain changes in NAFLD, there are limited comparative studies investigating the differences between hepatic steatosis and fibrosis, despite the progressive nature of the disease. Moreover, focused research on which specific brain regions are most vulnerable to liver disease remains largely lacking (Jiang et al. 2023).

To demonstrate the regional brain structural deviation in hepatic steatosis and fibrosis in terms of brain aging, we sought to adopt a normative modeling approach. Neuroanatomical normative modeling is an emerging technique used in neuroscience to capture individual‐level variability in the brain, making it applicable to various research questions (Verdi et al. 2023). This approach allows for statistical inferences about an individual's expected normative distribution or developmental trajectory over time. Specifically, it models the relationship between neurobiological variables (e.g., cortical thickness [CT]) and covariates (e.g., demographic factors such as age and sex), enabling the mapping of variation centiles across a cohort, represented as *Z*‐scores (Jiang et al. 2023; Rutherford, Kia, et al. 2022). It is particularly useful for identifying deviations from typical brain aging trajectories. It also demonstrates greater effect sizes, indicating higher sensitivity. In a recent study, this approach yielded the strongest statistically significant outcomes for group difference testing and classification tasks compared to raw data (Rutherford et al. 2023). Furthermore, normative modeling is well suited for evaluating variability in neuroanatomical presentations (Verdi et al. 2023).

In this study, we explore the different patterns of variation in CT in the brains of patients with hepatic steatosis and fibrosis using neuroanatomical normative modeling. Our primary goal was to quantify the spatial patterns of neuroanatomical heterogeneity by assessing CT in these patients. We aimed to determine deviations from normative values for each brain region, identify statistical outliers, and identify the group differences.

## 2 Materials and Methods

### 2.1 Patient Selection

We retrospectively enrolled 1122 men and women aged 20–88 years who underwent a comprehensive health check‐up program between January 2019 and December 2022 at Seoul National University Hospital Health Promotion Center in the Republic of Korea. Each participant underwent laboratory testing, anthropometric measurements, a questionnaire assessment, and a brain MRI scan. The laboratory tests included fasting glucose, hemoglobin A1c (HbA1c), total cholesterol, triglycerides (TG), high‐density lipoprotein cholesterol (HDL‐C), aspartate aminotransferase (AST), alanine aminotransferase (ALT), gamma‐glutamyl transferase (GGT), uric acid, and high‐sensitivity C‐reactive protein (hs‐CRP), as well as hepatitis B virus surface antigen (HBsAg), anti‐hepatitis C virus (anti‐HCV), and prothrombin time. Venous blood samples were collected from all participants before 10:00 a.m. after fasting for 12 h overnight. All biochemical analyses were conducted in the same laboratory using standard procedures (Lee et al. 2010).

**FIGURE 1:**
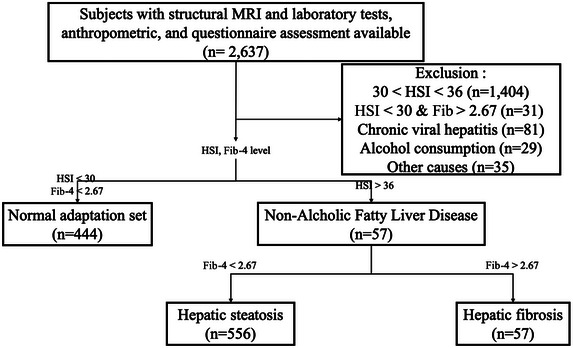
**Patient inclusion/exclusion criteria**. HSI, hepatic steatosis index.

We determined hepatic steatosis and hepatic fibrosis using the hepatic steatosis index (HSI) and fibrosis‐4 (Fib‐4) score, respectively. HSI is a simple, efficient screening tool for NAFLD first based on health check‐up subjects. HSI had an area under receiver‐operating curve of 0.812 (95% confidence interval, 0.801–0.824). At the value of < 30.0, HSI ruled out NAFLD with a sensitivity of 93.1% (Lee et al. 2010), and we used the cut‐off in this study. Fib‐4 score is a validated marker of liver fibrosis of multiple etiologies and a first‐line tool for risk stratification in clinical practice (Jiang et al. 2023; Sterling et al. 2006). Following earlier studies (Jiang et al. 2023; McPherson et al. 2017), participants with a Fib‐4 score > 2.67 were identified as having a high risk of advanced liver fibrosis. Participants with seropositivity for HBsAg or anti‐HCV antibody and excessive alcohol consumption (> 20 g/day) were excluded (Figure 1).

### 2.2 MRI Acquisition

MRI scans were performed using 3.0 T MR scanners (Discovery MR750w, GE Healthcare, Milwaukee, WI) with a 24‐channel head coil. Volumetric T1‐weighted images were acquired with a 3D fast spoiled gradient‐echo pulse sequence using the following parameters: repetition time of 8.5 ms, echo time of 3.2 ms, inversion time of 450 ms, flip angle of 12°, field of view of 256 × 256 mm, acquisition matrix of 256 × 256, number of excitations of 1, slice thickness of 1 mm, and between 154 and 172 slices depending on head size, all in the sagittal plane, with a voxel size of 1 mm^3^. Subjects were excluded if they had prior brain defects, such as lacunar infarctions larger than 3 mm (Loos et al. 2018). The remaining participants were categorized into three groups on the basis of their HSI and Fib‐4 scores: the Normal adaptation group (both HSI and Fib‐4 scores within the normal range), the hepatic steatosis group (HSI > 36 and Fib‐4 < 2.67), and the hepatic fibrosis group (HSI > 36 and Fib‐4 > 2.67) (Figure 1).

### 2.3 Imaging Processing and Quantification

Cortical surface reconstruction was performed using the MPRAGE (T1) image of each participant with the FastSurfer pipeline (https://github.com/Deep‐MI/FastSurfer) (Henschel et al. 2020). CT estimates were extracted for each participant from their respective FastSurfer output folders, then merged and formatted into a CSV file. The CT for each region in the Destrieux atlas was extracted using the aparcstats2table function (Rutherford et al. 2023). To assess the quality of reconstructions, we computed the Euler index (Bethlehem et al. 2020; Rosen et al. 2018). High‐quality scans were defined on the basis of previous studies: the absence of artifacts such as ghosting or ringing, successful completion of FastSurfer surface reconstruction, and an Euler number (a proxy for scan quality) calculated by FastSurfer that was below the set threshold (rescaled Euler < 10) (Kia et al. 2022).

### 2.4 Normative Model Formulation

Normative models were estimated using the Predictive Clinical Neuroscience toolkit (https://github.com/predictive‐clinical‐neuroscience/PCNtoolkit‐demo) (Rutherford, Kia, et al. 2022). For the structural data, we utilized a publicly available repository of pre‐trained normative models, which had been estimated using approximately 58,000 subjects from multiple datasets with a warped Bayesian linear regression algorithm (Fraza et al. 2021). Because our data did not originate from the same scanning sites as the training set, we need to account for the site effect, including ethnicity. The transferring process is done by first applying the warp parameters estimated on the training data to the new dataset, adjusting the mean and variance in the latent Gaussian space (Rutherford, Fraza, et al. 2022). We then adapted the estimates to our specific context using a modified transfer learning approach available at https://colab.research.google.com/github/predictive‐clinical‐neuroscience/PCNtoolkit‐demo/blob/main/tutorials/BLR_protocol/transfer_pretrained_normative_models.ipynb to remain unbiased. Covariates, including age, sex, data quality metric (Euler number), and scanning site, were included (Rutherford et al. 2023). The explained variance, mean standardized log loss, skewness, and kurtosis metrics were used to assess model fit (Figure S1). The outputs of the normative modeling process included a deviation score (*Z*‐score) for each brain region for every subject. These deviation scores indicate an individual's standing relative to the population: A positive deviation score indicates greater CT than average, whereas a negative score indicates less than average. The resulting *Z*‐scores were used as input features for the subsequent analyses.

### 2.5 Statistical Analysis

#### 2.5.1 Group CT Differences

We performed mass univariate group difference testing (hepatic steatosis vs. fibrosis) across all 148 brain regions. We estimated and ran two‐sample independent *t*‐tests on the data using MATLAB 2020b (The MathWorks, Inc., Natick, MA). Following a previous study, brain regions were considered significant if they survived multiple comparison correction using a false discovery rate (FDR)‐corrected *p* value of < 0.05, applied via the MATLAB function “mafdr” (Benjamini and Hochberg 1995; Verdi et al. 2023).

#### 2.5.2 Outlier Definition and Statistics

To reveal the difference in brain heterogeneity between hepatic steatosis and fibrosis, we identified the cortical areas with extreme deviation. Outliers were identified for each region, defined as *Z* > 1.96 and *Z* < −1.96 (corresponding to the top and bottom 2.5% of the normative distribution). The number of outliers was summed across 148 regions for each participant to give a total outlier count (tOC) across regions. We further categorized this count into the number of positive outliers (tOC‐pos) and negative outliers (tOC‐neg). Furthermore, we compared the number of outliers between groups to explore the difference in cortical heterogeneity. In addition, we looked for the correlation between tOC and clinical variables, such as HbA1c, hs‐CRP, and body mass index (BMI).

## 3 Results

### 3.1 Participants

A total of *n* = 1057 T1‐weighted MRI scans were evaluated, with 444 participants with normal HSI and Fib‐4 scores used as an adaptation set (Table 1). The hepatic steatosis group comprised a higher proportion of males and is younger than the hepatic fibrosis group.

**TABLE 1:** Clinical characteristics of study population.

	Normal (adaptation data) (*n* = 444)	Hepatic steatosis (*n* = 556)	Hepatic fibrosis (*n* = 57)	*p* value^*^	Effect size^a^
Age (years)	58.6 ± 9.42	61.6 ± 9.34	66.4 ± 7.94	< 0.01	0.52
Gender (male:female)	154:290	294:160	31:26	< 0.01	0.48
Body mass index (kg/mm^2^)	21.4 ± 2.2	27.2 ± 3.8	25.4 ± 3.1	< 0.01	0.46
HbA1c		5.9 ± 0.8	6.0 ± 0.8	0.62	0.07
hs‐CRP		0.22 ± 0.50	0.35 ± 0.58	0.17	0.18
tOC‐neg		2.2 ± 3.4	1.3 ± 2.0	0.06	0.23
tOC‐pos		4.2 ± 6.2	6.3 ± 10.3	0.02	0.26

*Note*: Data are mean ± standard deviation.

Abbreviations: HbA1c, hemoglobin A1c; hs‐CRP, high‐sensitivity C‐reactive protein; tOC‐neg, total negative outlier count; tOC‐pos, total positive outlier count.

^a^ Cohen's *d* for continuous variables and Cramer's *V* for categorical variables.

* *t*‐test test for continuous variables and chi‐square test for categorical variables, compared between hepatic steatosis and fibrosis groups.

### 3.2 CT Outliers in NAFLD: Regional Heterogeneity Analysis

NAFLD patients showed an increased number of both positive and negative outliers compared to the normal adaptation test set. Within the NAFLD group, patients with hepatic fibrosis had a higher proportion of CT outliers than those with hepatic steatosis.

**FIGURE 2:**
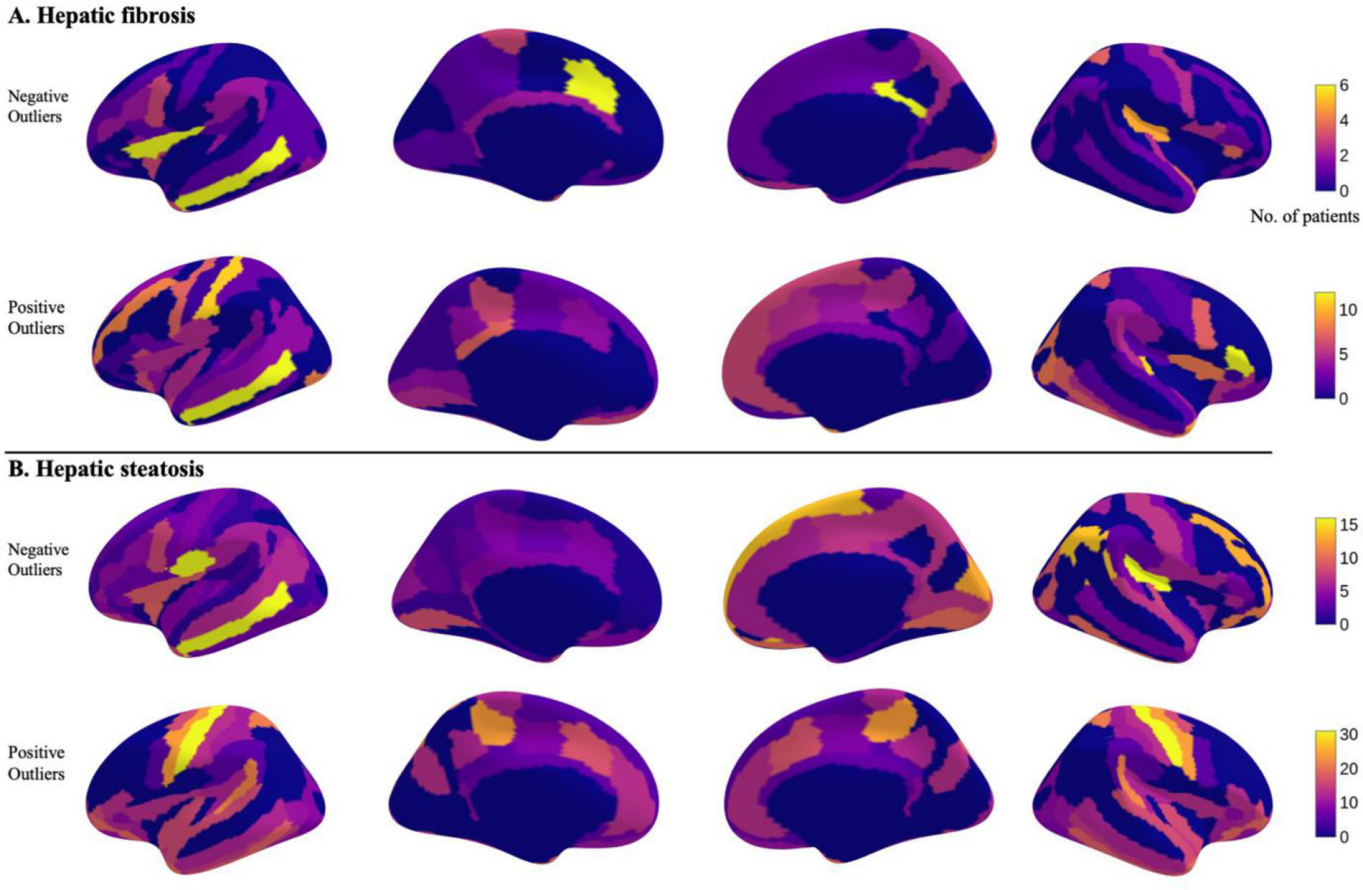
**Regional maps of heterogeneity**. Extreme deviations, separated into positive outliers and negative outliers, are summarized for each group (panel A : Hepatic steatosis, panel B : Hepatic fibrosis). For each brain region, the number of subjects with an extreme deviation in that region is counted.

The number of tOC‐pos differed significantly between groups, with hepatic fibrosis patients showing a greater mean number of thicker outliers than those with steatosis (6.3 ± 10.3 vs. 4.2 ± 6.2, respectively, *p* = 0.02, Table 1). The distribution of outliers is depicted in Figure 2, highlighting that both positive and negative outliers are more frequently found in the temporal and central cortex across both hepatic steatosis and fibrosis, indicating shared heterogeneity within NAFLD. Furthermore, when examining the correlation between clinical variables (HbA1c, hs‐CRP, and BMI) and tOC, no significant relationships were found, with correlation coefficients ranging from *r* = −0.0419 to 0.0274.

### 3.3 Mass Univariate Analysis of CT: Identifying Key Regions in NAFLD

**FIGURE 3:**
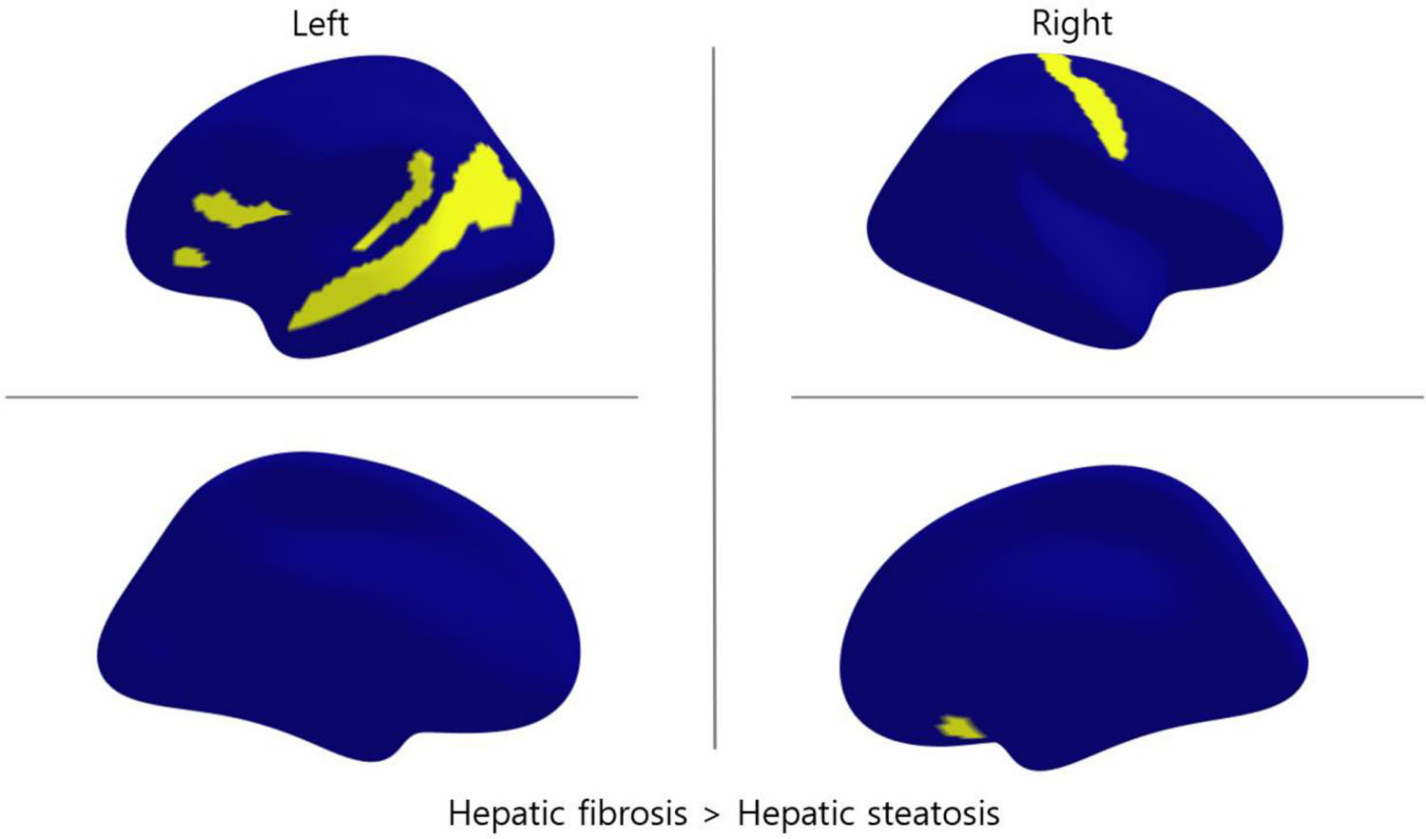
**Mass univariate analysis for group comparison between hepatic steatosis and fibrosis among NAFLD**. Significant group differences in cortical thickness deviation scores were observed after applying multiple comparison corrections. The hepatic fibrosis group had six areas of higher cortical thickness deviation scores. NAFLD, non‐alcoholic fatty liver disease.

The mass univariate test of group differences in *Z*‐scores of CTs across 148 brain regions identified 6 significant areas after FDR correction: lt_inferior_frontal_orbital, lt_lat_fiss_ant_hor, lt_sup_tem_sulcus, lt_superior_temporal_planum, rt_central, and rt_subcallosal. Against our hypothesis of hepatic fibrosis having a lower CT, all of the detected areas showed higher thickness in hepatic fibrosis groups (Figure 3 and centiles of variation plot in Figure S2). Comparing the raw CT for sensitivity analysis, no significant CT differences were found between the two groups.

## 4 Discussion

In this study, we used neuroanatomical normative modeling to examine different patterns of variation in CT among patients with hepatic steatosis and fibrosis. This approach allowed us to create an individual metric of neuroanatomical heterogeneity (i.e., tOC). Using normative modeling with a large dataset transferred to ours, we discovered a tendency of increased CT in patients with hepatic fibrosis than steatosis, concerning one's age and sex.

The results were somewhat counterintuitive. Given that hepatic steatosis progresses to fibrosis, a reduction in brain volume would seem more expected (Figure 3). In addition, previous neuropathological studies have reported that liver cirrhosis involves a loss of brain parenchyma (Chen et al. 2020; Kril and Butterworth 1997). However, this is not the first case reporting increased CT investigating the liver–brain axis (Lin et al. 2024). For example, Lin et al. (2024) reported that NAFLD causes an increase in the precuneus and suggested that this may be linked to the brain's hypoxic and inflammatory response, which leads to cellular edema or compensatory hypertrophy. Another possible explanation is the survivorship bias (Ellenberg 2014). Bias occurs when researchers focus on individuals who have passed a selection process while ignoring those who have not. Evidence suggests that hepatic steatosis, particularly when accompanied by fibrosis, is associated with a higher risk of adverse outcomes such as liver‐related mortality. Fibrosis is also one of the strongest predictors of long‐term mortality in NAFLD (Jeong 2020), and fibrosis is one of the most significant features of NAFLD linked to long‐term mortality (Angulo 2010). In addition, Chen et al. (2020) discovered that greater gray matter volume in the left middle and superior temporal areas predicts a lower likelihood of hepatic encephalopathy in liver cirrhosis. On the basis of the age of the hepatic fibrosis group being older than steatosis in our subjects, we may suggest that those subjects with thicker brain cortices survived longer than those not.

A notable finding is the shared heterogeneity in the temporal cortices, particularly the left middle temporal gyrus (Figure 2, first column). Variation in the temporal cortex has been observed in other disease etiologies that utilize the normative modeling approach. For example, a recent study used normative modeling to estimate neuroanatomical heterogeneity within an Alzheimer's disease cohort and reported variations in atrophy within the temporal regions (Pinaya et al. 2021; Verdi et al. 2023). In another recent study on serum liver function markers and brain, Chen et al. (2021) found that serum ALT levels were linked to brain perfusion, with higher ALT being associated with reduced blood flow in the middle temporal gyrus. Given that individuals with NAFLD exhibit lower cognitive performance across several domains (Kjærgaard et al. 2021), our findings of shared heterogeneity in the temporal cortex and Alzheimer's disease and NAFLD, although speculative, may suggest a link among hepatic dysfunction, neuroanatomical heterogeneity, and cognitive decline.

Lastly, we emphasize the advantages of using the normative modeling approach, which was the primary reason for selecting this method. Rutherford, Fraza, et al. (2022) have highlighted several key benefits of this approach. First, utilizing a large lifespan dataset enhances anatomical precision, which is crucial for distinguishing between conditions. Second, normative models provide flexibility by accommodating non‐Gaussian distributions and enabling seamless transfer to new sites. In our study, this approach proved particularly valuable, as a mass univariate comparison of raw CT did not reveal significant differences between the two groups. This finding aligns with previous studies demonstrating that normative modeling enhances sensitivity in detecting subtle neuroanatomical variations (Rutherford et al. 2023).

We also acknowledge several limitations. First, we exclude individuals with excessive alcohol consumption. However, some researchers have emphasized that patients with fatty liver disease should be evaluated regardless of etiology, including alcohol consumption, due to their overlapping features (Åberg and Färkkilä 2020). Furthermore, recent studies have identified alcohol dependence–related cortical changes, underscoring the importance of distinguishing NAFLD from AFLD in brain studies. Alcohol use may act as a potential confounder in the brain–liver relationship (Park et al. 2021). Second, due to the cross‐sectional study design, we cannot infer temporal relations between the hepatic steatosis progress to fibrosis and brain imaging markers. Third, although we adjusted for important potential confounders, we cannot rule out the possibility of residual confounding (e.g., genetic factors or additional clinical measurements). In addition, the baseline normative modeling used in our study was developed primarily on the basis of a Western population. Although we corrected for site noise and adaptation set, there may still be potential bias from different ethnicities, which could serve as a confounding factor. Fourth, to meet the diagnostic criteria for NAFLD, several rare liver diseases (e.g., Wilson's disease, hemochromatosis, and primary biliary cirrhosis) need to be identified. However, we were unable to do so due to a lack of medical information. Although these liver diseases are rare, some participants with undiagnosed liver conditions may still have been included, which could influence our findings. Lastly, we did not utilize imaging evidence to diagnose hepatic steatosis. However, we believe that HSI is a validated marker for diagnosing hepatic steatosis (Lee et al. 2010).

## 5 Conclusion

In conclusion, the results of this study support a possible link between liver disease and brain aging, specifically suggesting that hepatic fibrosis tends to be associated with greater CT compared to hepatic steatosis. Future research is needed to investigate the specific mechanisms underlying our findings in this cross‐sectional cohort.

## Author Contributions

**Minchul Kim**: conceptualization, data curation, formal analysis, visualization, writing – original draft, writing – review and editing. **Inpyeong Hwang**: methodology, conceptualization, investigation, validation, supervision, resources, funding acquisition, writing – original draft. **Kyu Sung Choi**: conceptualization, methodology, supervision, funding acquisition, resources, writing – original draft. **Junhyeok Lee**: investigation, project administration. **Minjung Ryu**: investigation, project administration. **Jung Hyun Park**: investigation, project administration. **Joon Ho Moon**: methodology, conceptualization.

## Ethics Statement

This retrospective study was approved by the Institutional Review Board (IRB No. 2203‐178‐1313), and informed consent was waived.

## Consent

The authors have nothing to report.

## Conflicts of Interest

The authors declare no conflicts of interest.

### Peer Review

The peer review history for this article is available at https://publons.com/publon/10.1002/brb3.70466

## Supporting information



Supporting Information

## Data Availability

Data generated or analyzed during the study are available from the corresponding author by request.
