# Supplementary material for: Normative Modeling Reveals Age‐Atypical Cortical Thickness Differences Between Hepatic Steatosis and Fibrosis in Non‐Alcoholic Fatty Liver Disease
Source: Brain Behav. 2025 Apr 7;15(4):e70466. doi: 10.1002/brb3.70466 (PMC11975609; doi:10.1002/brb3.70466)
Supplement: Supplementary file 1 — Supporting Information [file BRB3-15-e70466-s001.docx]

**Supplementary Results**

**Figure S1.**


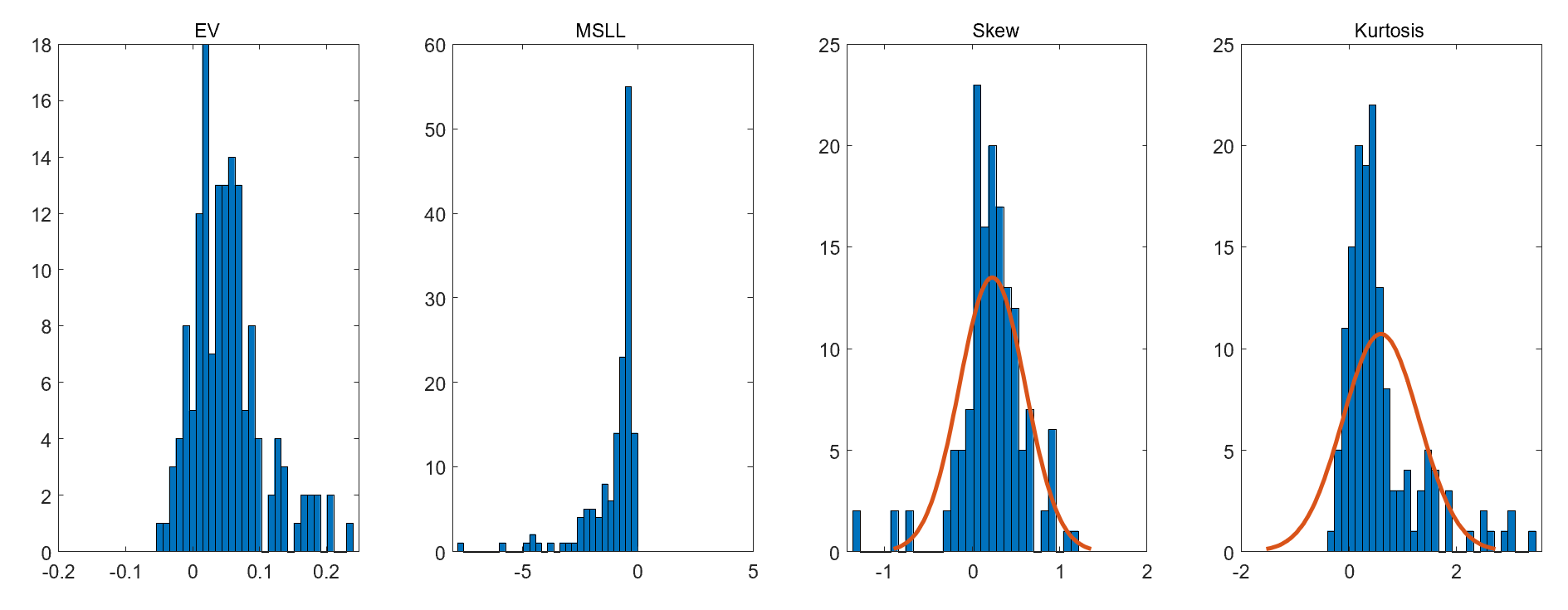


Normative model evaluation metrics. The distribution across all evaluation metrics in the test set. Higher explained variance (EV), more negative mean squared log-loss (MSLL), and normally distributed skew and kurtosis correspond to better model fit.

**Figure S2.** **Centiles of variation plot**


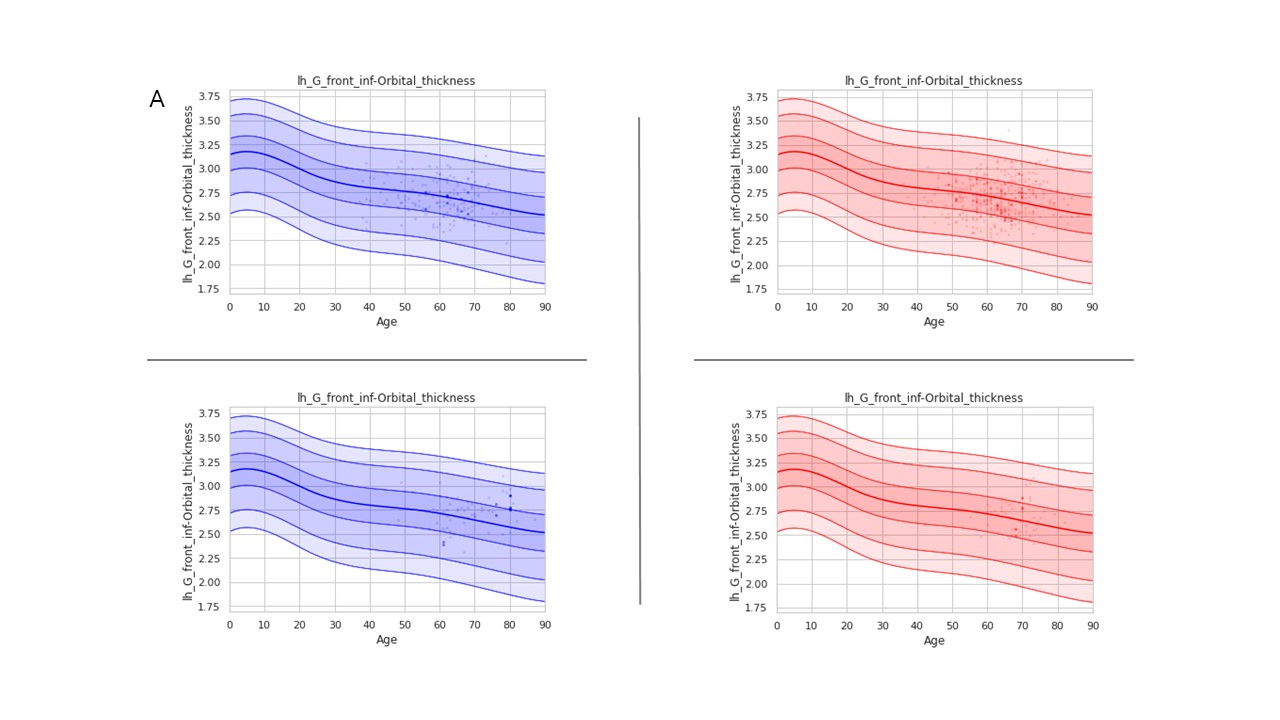

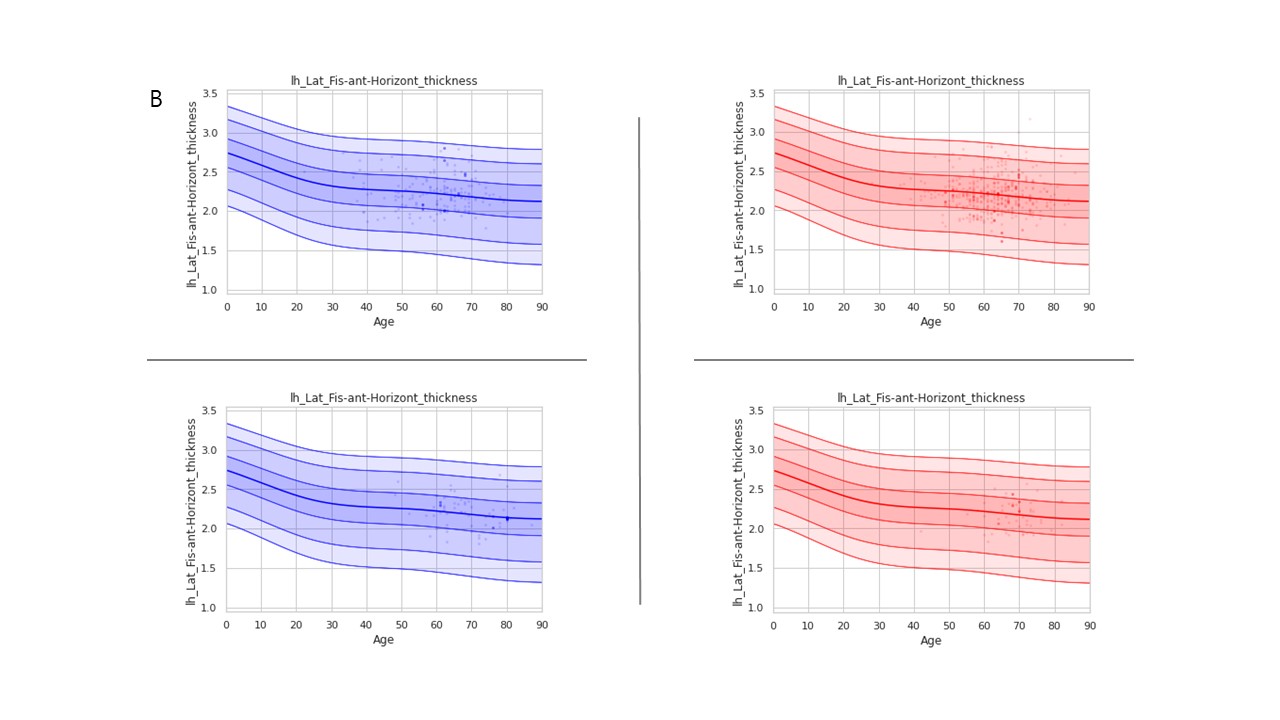


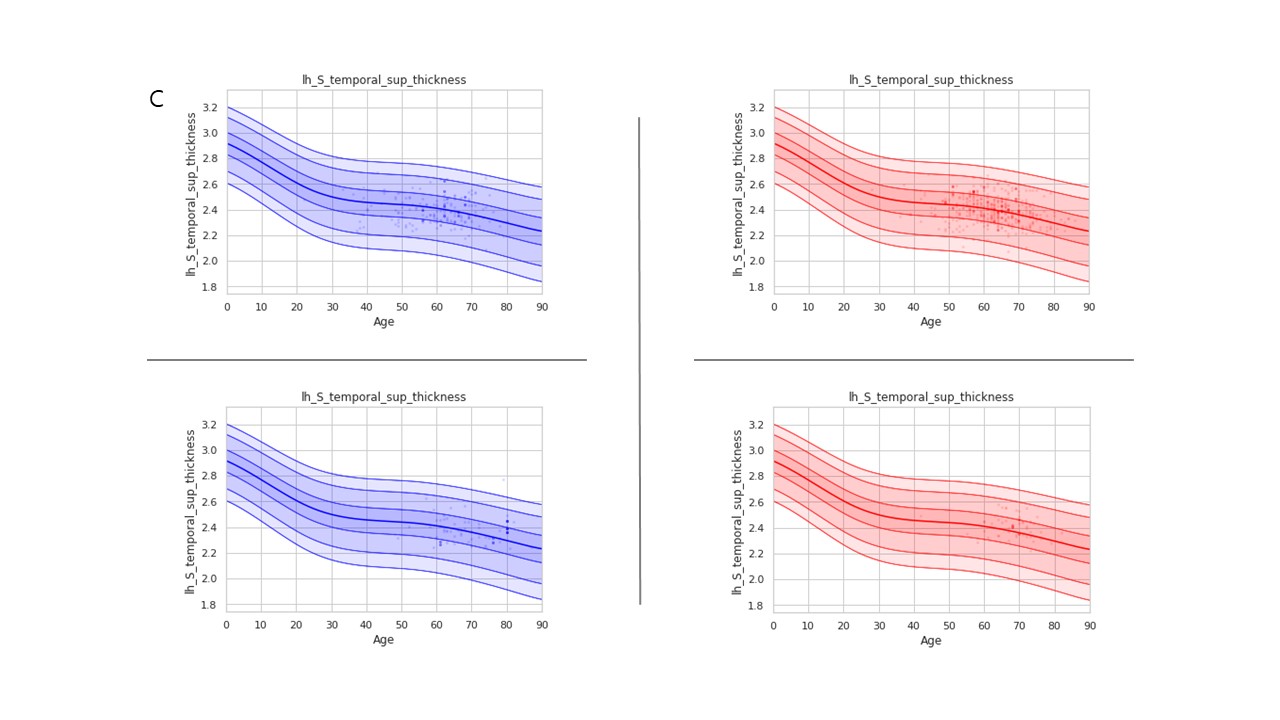


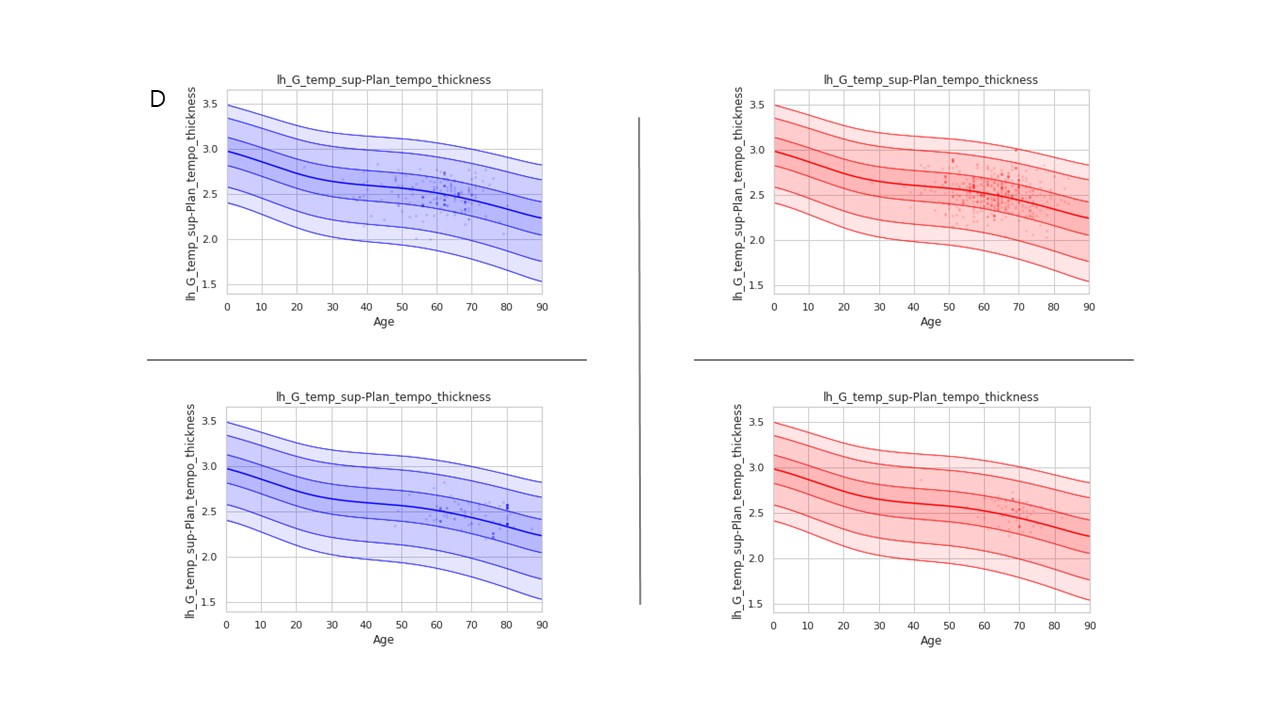

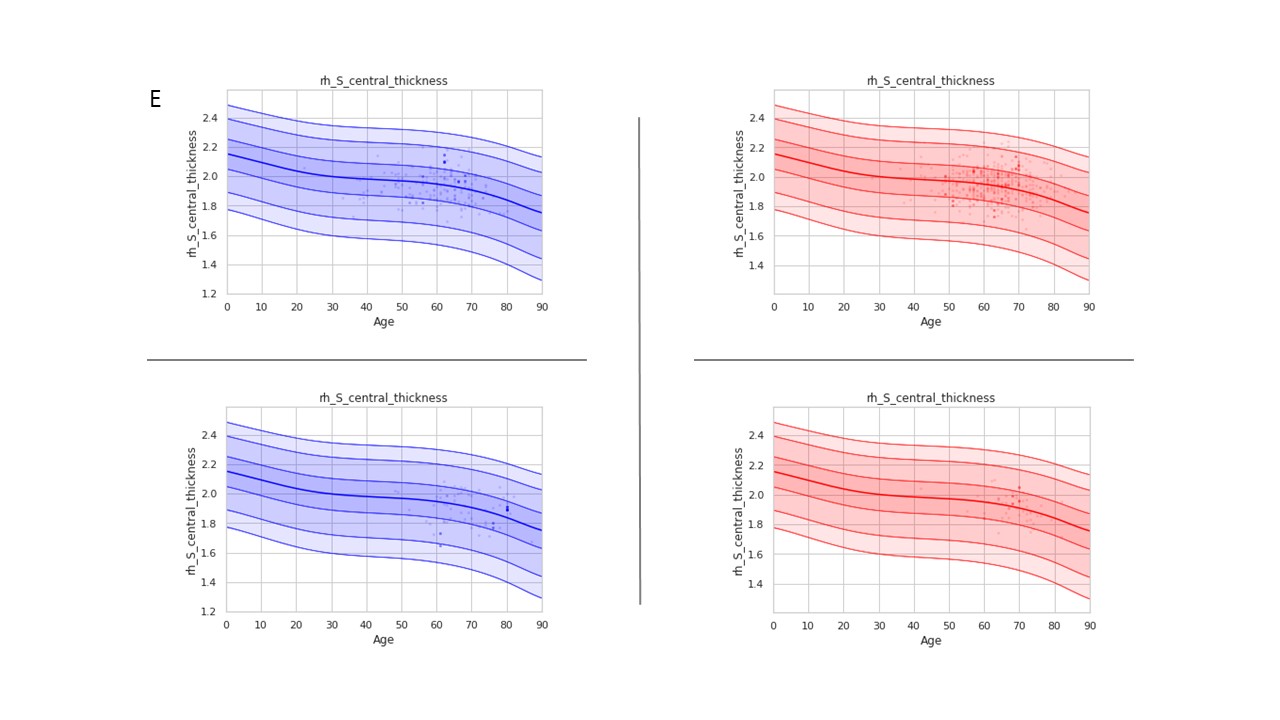

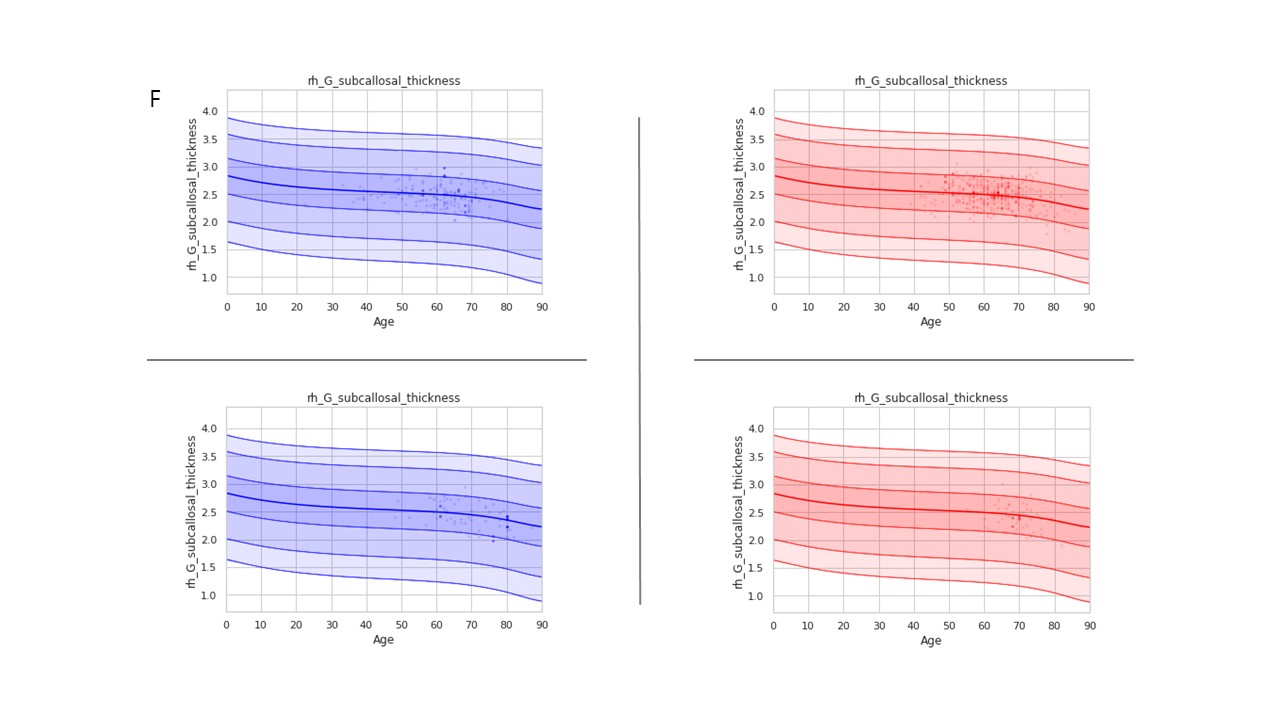
Cortical thicknesses of each brain region are predicted from age (x-axis) using a training set consisting of multisite structural MRI from neurotypical controls and a test set consisting of hepatic steatosis (*upper row*), and hepatic fibrosis (*lower row*) patient groups. Every dot indicates the deviation score for a single individual from normal aging.
